# Evidence for transcript-specific epigenetic regulation of glucocorticoid-stimulated skeletal muscle 11β-hydroxysteroid dehydrogenase-1 activity in type 2 diabetes

**DOI:** 10.1186/1868-7083-4-24

**Published:** 2012-12-17

**Authors:** Warrick J Inder, Varuni R Obeyesekere, Christina Jang, Richard Saffery

**Affiliations:** 1 Department of Endocrinology and Diabetes, St Vincent’s Hospital, 41 Victoria Parade, Fitzroy, VIC 3065, Australia; 2 Department of Diabetes and Endocrinology, Princess Alexandra Hospital, Ipswich Road, Woolloongabba, QLD 4102, Australia; 3 School of Medicine, University of Queensland, 288 Herston Road, Herston, QLD, 4006, Australia; 4 Centres for Health Research, Princess Alexandra Hospital, Ipswich Road, Woolloongabba, QLD, 4102, Australia; 5 Developmental Epigenetics, Murdoch Children’s Research Institute and Department of Paediatrics, University of Melbourne, Royal Children’s Hospital, Flemington Road, Parkville, VIC, 3052, Australia

**Keywords:** 11β-Hydroxysteroid dehydrogenase-1, Cortisol, Diabetes, Epigenetics, DNA methylation

## Abstract

**Background:**

The enzyme 11β-hydroxysteroid dehydrogenase type 1 (11βHSD1) converts inactive cortisone into active cortisol in insulin target tissues. In people with type 2 diabetes, skeletal muscle (SkM) 11βHSD1 is upregulated by the potent glucocorticoid dexamethasone. The *HSD11B1* gene has two promoters designated P1 and P2. CCAAT/enhancer-binding protein beta (C/EBPβ) is known to regulate expression of 11βHSD1 via the P2 promoter. In this study, we investigated the potential role of altered DNA methylation of the P1 and P2 promoters in the observed dexamethasone-induced upregulation of SkM 11βHSD1 oxoreductase activity in human diabetic subjects. SkM biopsies from 15 people with type 2 diabetes were collected before and after treatment with oral dexamethasone 4 mg/day for 4 days and SkM 11βHSD1, C/EBPβ and P1 and P2 promoter region mRNA levels were measured by quantitative RT-PCR. 11βHSD1 oxoreductase activity was quantified by measuring the conversion of radiolabeled ^3^H-cortisone to cortisol by thin layer chromatography. Analysis of *HSD11B1* promoter methylation (P1 and P2) was performed using Sequenom MassARRAY EpiTYPER analysis.

**Results:**

Dexamethasone treatment resulted in a significant increase in 11βHSD1 mRNA levels (*P* = 0.003), oxoreductase activity (*P* = 0.017) and C/EBPβ mRNA (*P* = 0.015), and increased expression of both the P1 (*P* = 0.008) and P2 (*P* = 0.016) promoter regions . The distal P1 promoter region showed a significant reduction in methylation following dexamethasone (*P* = 0.026). There was a significant negative correlation between the change in methylation at this site and the increment in 11βHSD1 oxoreductase activity (*r* = −0.62, *P* = 0.014).

**Conclusions:**

Our findings of reduced methylation in the *HSD11B1* P1 promoter in association with increased 11βHSD1 oxoreductase activity implicate complex multi-promoter epigenetic mechanisms in the regulation of 11βHSD1 levels in SkM.

## Background

11β-Hydroxysteroid dehydrogenase-1 (11βHSD1) is a bi-directional enzyme, most abundantly expressed in glucocorticoid target tissues such as the liver and adipose [1]. 11βHSD1 displays predominant oxoreductase activity in intact cells, generating the biologically active glucocorticoid cortisol from the inactive form, cortisone [2]. In adipose tissue, several studies show increased 11βHSD1 expression and oxoreductase activity, suggesting that differential elevation of glucocorticoid in this tissue may be involved in the pathogenesis of central obesity [3]. Skeletal muscle (SkM) is a major site of insulin resistance and accounts for the majority of peripheral glucose disposal [4]. We have previously demonstrated that 11βHSD1 is present and biologically active in SkM of both diabetic and nondiabetic subjects [5].

Previous *in vitro* studies in human myoblasts have demonstrated that addition of glucocorticoid upregulates 11βHSD1 mRNA levels and activity [6]. This is consistent with other studies in a variety of human tissues including skin fibroblasts [7], amnion fibroblasts [8], and omental adipose stromal cells [9] that implicate glucocorticoids in 11βHSD1 upregulation. Our own data in people with type 2 diabetes showed that orally administered dexamethasone increases SkM 11βHSD1 oxoreductase activity [5], but this response was not observed in nondiabetic subjects.

Dexamethasone increases 11βHSD1 mRNA levels and activity in cultured chorionic trophoblasts, an effect that is blocked by the glucocorticoid receptor antagonist RU486 [10]. Co-localization of 11βHSD1 and the glucocorticoid receptor was demonstrated [10]. This indicates that the stimulatory effect of glucocorticoids on 11βHSD1 is likely to be mediated via the glucocorticoid receptor. Glucocorticoids appear to act in an autocrine manner, stimulating the expression of 11βHSD1, which in turn increases conversion of cortisone to active cortisol, a so-called positive feed-forward mechanism [11].

**Figure 1:**
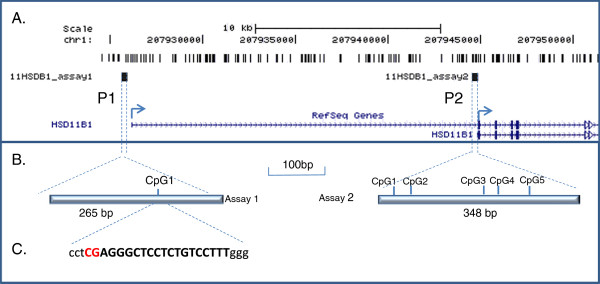
**Location of the *HSD11B1 *promoter methylation assays used in this study. (A) **Genome coordinates on chromosome 1, location of individual CpG dinucleotides (dashes) and methylation assay location (black boxes) relative to the 11β-hydroxysteroid dehydrogenase-1 *HSD11B1 *gene (vertical blue dashes, exons; arrowed lines, introns – according to the UCSC genome browser hg_18 assembly). Arrows denote transcriptional direction. P1 and P2 refer to two independent promoters examined in this study. **(B) **Higher resolution view of methylation assay 1 (containing a single CpG site adjacent to a conserved putative glucocorticoid response element (GRE)) and assay 2 (containing five CpG sites, three of which were amenable to measurement of methylation levels by Sequenom). **(C) **Sequence of conserved GRE located within the *HSD11B1 *P1 region (uppercase letters) relative to the CpG site assayed in this study (red lettering).

The human genome contains two annotated transcription start sites for the *HSD11B1* gene (encoding 11βHSD1), which are potentially regulated in an independent manner in different tissues (Figure 1). However, mechanisms underlying regulation of this gene remain poorly understood. Several studies have demonstrated the importance of the CCAAT/enhancer-binding protein (C/EBP) transcription factors [11-16] in *HSD11B1* expression from the proximal P2 promoter. In human A549 lung epithelial cells, C/EBPβ has been shown to mediate the induction of 11βHSD1 by glucocorticoids [13], whereas in human amnion fibroblasts C/EBPα rather than C/EBPβ was involved [11]. However, inhibition of C/EBP is insufficient to completely block glucocorticoid stimulation of 11βHSD1 activity, at least in some cells [11].

DNA methylation is an epigenetic process that plays a pivotal role in the regulation of gene expression in both a temporal and spatial manner throughout development [17]. Methylation of DNA in vertebrates occurs almost exclusively in CpG dinucleotides, often clustered in gene promoter regions. Promoter methylation is generally associated with reduced gene activity. Changes in promoter methylation may occur in a dynamic fashion with measured periodicity of as little as 100 minutes [18,19]. The observed induced 11βHSD1 enzyme activity following glucocorticoid treatment may therefore potentially be associated with a reduction in *HSD11B1* promoter methylation. Each of the two annotated *HSD11B1* promoters contains CpG sites, one of which (within the distal P1 promoter) overlaps a conserved glucocorticoid response element (GRE; Figure 1).

In this study, we tested the hypothesis that the increased 11βHSD1 mRNA expression and enzyme activity seen in SkM post dexamethasone treatment is associated with a reduction in DNA methylation at the putative GRE of the *HSD11B1* P1 gene promoter in human diabetic subjects.

## Results

The subjects consisted of seven males and eight females with a median age 57 years (range 48 to 66 years). Overall they had moderately well controlled diabetes, with a median (25th to 75th percentile) HbA1c of 6.9% (5.8 to 7.8%). All subjects had some degree of glucocorticoid-induced hyperglycemia and insulin resistance following dexamethasone treatment, with a significant increase in fasting glucose, insulin and HOMA2-IR homeostasis model assessment (Table 1).

**Table 1:** Patient characteristics before and after dexamethasone 4 mg/day for 4 days

**Characteristic**	**Pre dexamethasone**	**Post dexamethasone**
Fasting glucose (mmol/l)	7.0 (6.1 to 9.8)	7.6 (6.4 to 12.9)^a^
Fasting insulin (mU/l)	13.1 (9.6 to 15.5)	15.8 (11.4 to 17.8)^a^
HOMA2-IR	2.0 (1.3 to 2.1)	2.4 (1.6 to 2.9)^b^
11βHSD1 mRNA (relative expression)	3.06 (1.81 to 4.61)	3.06 (2.70 to 6.11)^b^
11βHSD1 oxoreductase activity (% conversion)	11.3 (4.7 to 17.4)	15.5 (11.6 to 19.3)^a^
C/EBPβ mRNA (relative expression)	1.37 (0.32 to 2.46)	2.06 (0.85 to 3.11) ^a^
P1 promoter mRNA (relative expression, *n* = 11)	0.03 (0.02 to 0.04)	0.06 (0.03 to 0.22)^b^
P2 promoter mRNA (relative expression, *n* = 11)	0.83 (0.63 to 1.13)	1.40 (0.77 to 1.78)^a^

Data presented as median (25th to 75th percentile). The Wilcoxon signed-rank test examined the significance of the median difference between paired values. 11βHSD1, 11β-hydroxysteroid dehydrogenase-1; C/EBP, CCAAT/enhancer-binding protein; HOMA, homeostasis model assessment. *P *<0.05 compared with pre dexamethasone^a^. *P <*0.01compared with pre dexamethasone^b^.

**Figure 2:**
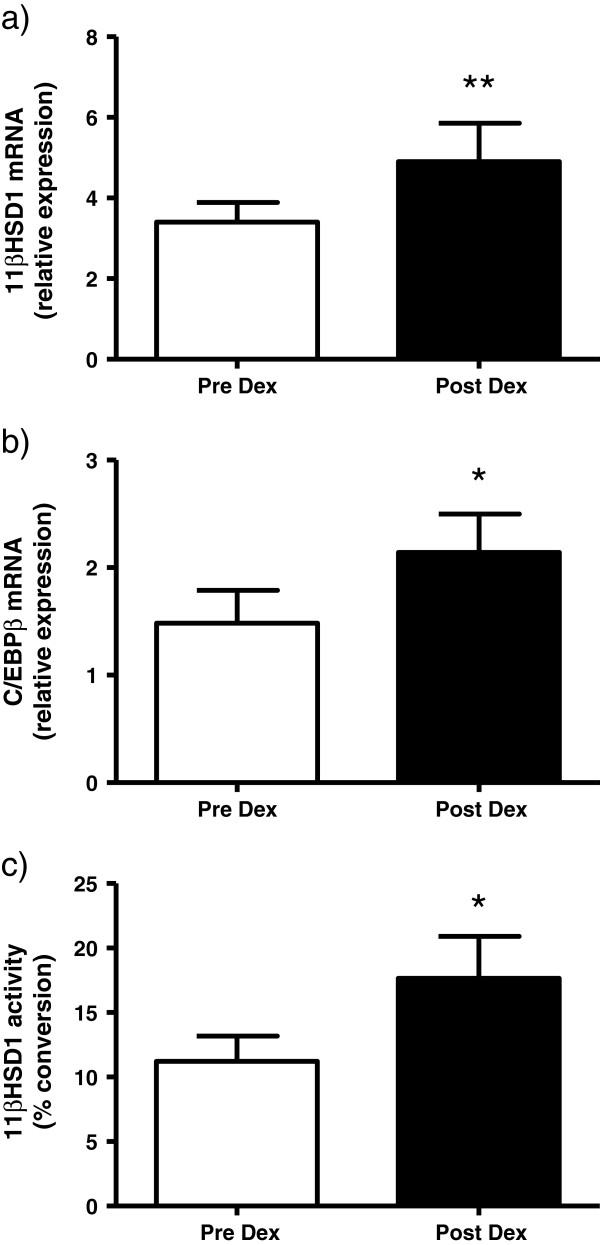
**Skeletal muscle 11β-hydroxysteroid dehydrogenase-1 mRNA, C/EBPβ and 11βHSD1 oxoreductase activity before and after dexamethasone. **Mean ± standard error of skeletal muscle **(a) **11β-hydroxysteroid dehydrogenase-1(11βHSD1) mRNA, **(b) **CCAAT/enhancer-binding protein (C/EBPβ) and **(c) **11βHSD1 oxoreductase activity before (□) and after (■ ) dexamethasone 4 mg/day for 4 days in 15 subjects with type 2 diabetes. *P <0.05, **P <0.01 by Wilcoxon signed-rank test.

Dexamethasone resulted in a significant increase in 11βHSD1 mRNA (*P* = 0.003), C/EBPβ mRNA (*P* = 0.015) and increased 11βHSD1 oxoreductase activity (*P* = 0.017) in SkM biopsies, as determined by the Wilcoxon signed-rank test, which examines the significance of the median difference between paired values (Table 1 and Figure 2). In the 11 subjects where sufficient cDNA remained for the determination of differential P1 and P2 promoter-associated expression, there was a significant twofold increase in the P1 promoter derived-transcript (*P* = 0.008) and to a lesser extent the P2 promoter-derived transcript (1.5-fold increase; *P* = 0.016).

**Figure 3:**
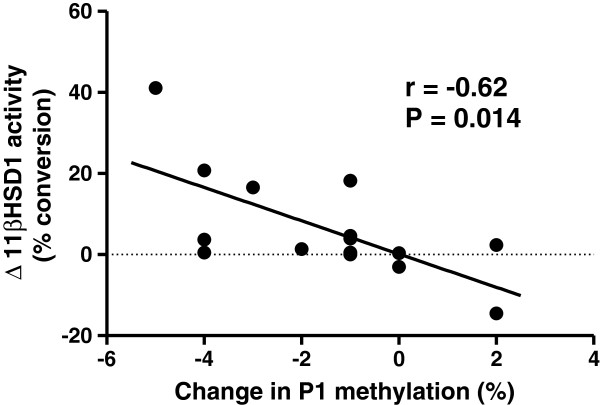
**Correlation between change in P1 promoter methylation and 11β-hydroxysteroid dehydrogenase-1 oxoreductase activity in skeletal muscle.***r *= −0.62, *P *= 0.014.

DNA methylation β-values obtained for *HSD11B1* promoters P1 (single CpG site) and P2 (3 CpG sites) are listed in Table 2. In the case of P2, the average methylation level was calculated from the three analyzable CpG sites. The distal P1 region, containing a putative GRE, showed a significant reduction in median methylation level, from 32% to 30%, following dexamethasone treatment (*P* = 0.026). Furthermore, there was a significant negative correlation via the Spearman test between the change in methylation at this site and the increment in 11βHSD1 oxoreductase activity (Figure 3, *r* = −0.62, *P* = 0.014). In the P2 promoter region, there was no significant change in total DNA methylation across the three sites tested (*P* = 0.093), although a consistent decrease in median methylation was observed at each site post dexamethasone treatment. There was not a significant correlation between the change in 11βHSD1 mRNA and P1 methylation.

**Table 2:** DNA methylation scores before and after dexamethasone 4 mg/day for 4 days

**Methylation site**	**Before dexamethasone (%)**	**After dexamethasone (%)**	***P *value**
P1 promoter CpG	32 (29 to 35)	30 (28 to 33)	0.026
P2 promoter (CpG mean)	42 (37 to 43)	38 (36 to 41)	0.093
(P2_CpG 2)	22 (16 to 24)	18 (17 to 22)	0.072
(P2 _CpG 3)	41 (33 to 44)	37 (35 to 42)	0.409
(P2 _CpG 4)	63 (58 to 66)	59 (56 to 62)	0.065

Data presented as median (25th to 75th percentile). Methylation scores (approximating % of alleles with cytosine methylation at specific CpG sites within a tissue sample) were obtained from the Mass Spec output using EpiTYPER v1.0.5 software (Sequenom Inc, San Diego, CA, USA). Only a single CpG site was present in the P1 promoter region tested, whereas three sites in close proximity were assessed in P2.

## Discussion

We have previously demonstrated that oral dexamethasone administered at a dose of 4 mg/day for 4 days increases 11βHSD1 oxoreductase activity in SkM of subjects with type 2 diabetes but not nondiabetic subjects [5]. We have now extended these findings by examining the association of the DNA methylation level in two annotated promoter regions with gene expression levels and enzyme activity in the diabetic group, which is subject to upregulation by dexamethasone. Evidence, primarily from accumulated expressed sequence tag data, suggests that the *HSD11B1* has two independent transcription start sites and associated promoters, designated P1 [14] and P2. In the mouse, the P1 promoter predominates in the lung and is independent of C/EBPα, while the P2 promoter predominates in the liver, adipose tissue and brain [14]. At present the predominant transcriptional start site in SkM remains unclear, although our data confirm that both transcripts are present. The P1 promoter region is CpG poor but contains a conserved putative GRE upstream of the transcriptional start site.

In this study we demonstrated that the dexamethasone-induced increase in overall 11βHSD1 mRNA, derived from both P1 and P2 promoters, and enzyme activity is associated with a significantly reduced level of DNA methylation at the putative GRE-associated CpG site in *HSD11B1* P1. Further, the extent of methylation reduction correlates significantly with the subsequent increase in 11βHSD1 enzyme activity. While a statistical association does not prove causality, the findings suggest that *HSD11B1* expression may be subject to dynamic epigenetic modulation in response to glucocorticoid status. This is further supported by the finding of significant upregulation of the P1 promoter-derived transcript following dexamethasone treatment. The absolute changes in methylation were small, but so were the changes in SkM 11βHSD1 activity in many of the subjects. Indeed in two subjects where a small decrease in 11βHSD1 activity was observed, P1 promoter methylation was increased or unchanged. Such a modest effect size is in accordance with other emerging studies in complex disease that routinely report small changes in DNA methylation in a case–control setting. As an example, Rakyan and colleagues identify small disease-associated methylation differences of 1.3 to 6.6%, which compare well with other multiple referenced studies in complex disease and our data [20]. They point out that this could be ‘the norm for complex disease associated epigenetic variation’ [20].

While methylation of a single CpG site was analyzed in the P1 promoter region, analysis of methylation of the CpG sites within the P2 region did not produce statistically significant changes following dexamethasone. The dose of dexamethasone utilized in this study is within the range used clinically, and completely suppresses the hypothalamic–pituitary adrenal axis, leading to the near absence of circulating cortisol. Dexamethasone induced insulin resistance in both diabetic and nondiabetic subjects and resulted in an elevation in fasting glucose, insulin and HOMA2-IR homeostasis model assessment in the current study.

P2-associated transcription of *HSD11B1* via the C/EBP transcription factors has previously been described [11]. The 11βHSD1 P2 promoter contains 10 C/EBP binding sites. The precise role of the different C/EBPs in the regulation of 11βHSD1 appears to be tissue specific. In HepG2 (hepatoma) cells, C/EBPα is the predominant regulator of 11βHSD1 expression, while C/EBPβ is only a weak activator in the absence of C/EBPα [15]. Both C/EBPα and C/EBPβ are required for basal transcriptional activity of 11βHSD1 in the mouse pre-adipocyte cell line 3T3-L1 [12]. Induction of C/EBPα shows a greater increase in basal 11βHSD1 expression, while C/EBPβ is the key factor mediating the increase in 11βHSD1 in response to cyclic AMP stimulation in these cells [12]. In human A549 lung epithelial cells, the stimulatory effect of dexamethasone on 11βHSD1 activity appears to be mediated by two mechanisms. Firstly, 11βHSD1 activity is dependent on the glucocorticoid receptor, since the co-administration of the glucocorticoid receptor antagonist RU486 blocks it; and secondly, the stimulatory effect of dexamethasone is indirect requiring new protein synthesis, since the glucocorticoid upregulation is also blocked by the protein synthesis inhibitor cycloheximide [13]. While a GRE lies between two key C/EBP binding sites in the P2 promoter, mutation of this GRE region did not appear to affect dexamethasone responsiveness [13]. C/EBPβ, but not C/EBPα or C/EBPδ, is a crucial mediator of the increased 11βHSD1 expression and activity following dexamethasone in A549 cells [13]. In contrast, in the human adipocyte cell line PAZ6, C/EBPα but not C/EBPβ or C/EBPδ was induced by dexamethasone [21].

We have shown here that humans administered exogenous dexamethasone exhibit a significant rise in SkM C/EBPβ mRNA levels consistent with the findings of Yang and colleagues [16]. The extent of the increase in C/EBPβ and 11βHSD1 in SkM was considerably less than observed by Sai and colleagues, who showed a 12-fold increase in 11βHSD1 mRNA levels in response to a threefold to fourfold increase in C/EBPβ in A549 lung epithelial cells [13]. Since our data demonstrated an increase in expression of both P1 and P2 promoters, we suggest that there may be a dual mechanism of dexamethasone-induced activation of SkM 11βHSD1 activity; via demethylation of the P1 promoter and through C/EBPβ stimulation of the P2 promoter.

In SkM, the precise mechanism(s) involved in glucocorticoid-induced activation of 11βHSD remain to be characterized. Which promoter (P1 or P2) is used in muscle to transcribe the *HSD11B1* gene in response to glucocorticoid was previously not known, although we have now shown that both transcripts are clearly detectable by specific RT-PCR. *In vitro* reporter experiments aimed at demonstrating functionality of the putative P1-associated GRE in appropriate cell models are required to confirm the functionality of this region in the observed glucocorticoid response. The P2-associated CpG sites are not associated with a putative GRE, suggesting that a reduction in methylation at the P2 promoter of *HSD11B1* is not associated with the increased SkM 11βHSD1 activity following dexamethasone administration.

The enzyme 11βHSD type 2 (11βHSD2), which converts cortisol to cortisone, is highly expressed in mineralocorticoid target tissues such as the kidney and colon, but is expressed at low levels in SkM [5]. There is evidence that 11βHSD2 is subject to epigenetic regulation, both at the level of DNA methylation but also of the histone modification profile [22]. Human subjects who developed hypertension when treated with prednisone had increased 11βHSD2 promoter methylation in peripheral blood mononuclear cells [23]. Our previous work demonstrated that SkM 11βHSD2 expression and activity was downregulated in the type 2 diabetic subjects [5]. This downregulation may thus possibly be associated with increased methylation of the 11βHSD2 promoter, but this was not the focus of the present study.

Methylation of DNA is a well-described mechanism of epigenetic modulation of gene expression. DNA methylation had been assumed not to be as dynamic as other epigenetic processes. However, recent studies have demonstrated that highly dynamic DNA methylation changes can occur even during the relatively short period of a single cell cycle in a number of human genes [13,19]. Other studies have examined epigenetic mechanisms in metabolic pathways in SkM of human subjects. Non-CpG methylation of the peroxisome proliferator-activated receptor γ coactivator-1α (PGC-1α) gene in myotubes has been associated with reductions in mitochondrial density in subjects with type 2 diabetes [24]. Furthermore, elevated DNA methylation of SkM PGC-1α is observed in normal birth weight nondiabetic subjects overfed a high-fat diet [25]. Our findings support the involvement of DNA methylation at the conserved putative GRE in the P1 11βHSD1 promoter in the regulation of glucocorticoid-induced *HSD11B1* gene activation in human SkM. Specifically, we find that the extent of the reduction in methylation of this GRE following dexamethasone correlates with the subsequent increment in SkM 11βHSD1 oxoreductase activity. While the putative GRE in the P1 11βHSD1 promoter may have a lesser role compared with C/EBP-stimulated P2 promoter activation in many tissues, our results suggest that even relatively small changes in methylation may influence the extent of the increment in SkM 11βHSD1 activity. However, this does not preclude the existence of a parallel response involving C/EBPβ action at the P2 promoter. Further functional studies are required to determine the relative contribution of these promoter regions and regulatory mechanisms to the overall regulation of the *HSD11B1* gene in SkM.

## Conclusion

This study has found a reduction in methylation of the putative GRE within the P1 promoter of *HSD11B1* that correlates with gene expression and the increase in 11βHSD1 oxoreductase activity in SkM following dexamethasone treatment. This observation implicates dynamic epigenetic remodeling in the regulation of glucocorticoid action via 11βHSD1.

## Methods

The experimental protocol was approved by the institutional Human Research Ethics Committee. Fifteen volunteers with type 2 diabetes gave written informed consent for participation. The subjects were treated by diet alone (*n* = 4) or with oral hypoglycemic agents (metformin alone, *n* = 2; a sulphonylurea alone, *n* = 2; or both, *n* = 7). No patient was using insulin or thiazolidinediones. Exclusion criteria included poorly controlled hypertension (systolic blood pressure ≥160 mmHg and/or diastolic blood pressure ≥90 mmHg), a history of liver or renal disease, and evidence of unstable angina or peripheral vascular disease. Data from 12 of these subjects were included in our previous study examining the effect of oral dexamethasone on 11βHSD1 and 11βHSD2 in diabetic and nondiabetic subjects [5]. Nondiabetic subjects were not included in this study because we had previously shown that the induction of 11βHSD1 oxoreductase activity did not occur in this group.

### Experimental design

After an overnight 10-hour fast, subjects had venous blood samples drawn between 08:00 and 08:30 hours for glucose, insulin and HbA1c. Body weight, body mass index and waist-to-hip ratio were determined for each subject.

After sedation with intravenous midazolam and administration of local anesthetic, biopsy of the vastus lateralis was performed using a 5 mm Bergstrom needle as previously described [26]. Fresh muscle was rapidly dissected of visible adipose and connective tissue and placed in DMEM for enzyme activity studies. Additional SkM from the same biopsy was snap-frozen in liquid nitrogen for mRNA analysis and epigenetic studies, with storage at −70°C. Within 28 days, subjects were administered oral dexamethasone (4 mg daily) for 4 days before repeat blood sampling and muscle biopsy. The subjects were clear that the dexamethasone was being administered for experimental rather than therapeutic purposes, and warned that dexamethasone would increase their blood glucose levels. They monitored their capillary blood glucose levels at least twice daily during dexamethasone therapy, and were contacted by an investigator on a daily basis.

### Laboratory assays

Plasma glucose was analyzed by a glucose oxidase method employing a YSI 1500 Sidekick analyzer (Yellow Springs Instrument Company, Yellow Springs, OH, USA), with coefficient of variation 2.4%. Plasma insulin was measured with a radioimmunoassay, with <1% cross-reactivity to proinsulin and sensitivity of 0.5 mU/l. Insulin antibodies were not present in any sample. HbA1c was measured by a high-performance liquid chromatography assay. Homeostasis model assessment was calculated using the computer program of Levy and colleagues [27].

### Quantitative real-time RT-PCR

Relative SkM mRNA levels for 11βHSD1 and C/EBPβ were determined using quantitative real-time RT-PCR (Taqman®, Applied Biosystems, Foster City, CA, USA ). Singleplex reactions were performed using 11βHSD1 specific primers with normalization to the endogenous control human 18s rRNA (Applied Biosystems, Foster City, CA, USA) as in our previous studies [5,26]. The RT-PCR product overlapped exons 2 and 3 of the *HSD11B1* gene. Relative 11βHSD1 mRNA levels were calculated using the ∆∆C_T_ methodology (using the formula 2^∆^^∆^^CT^). Human C/EBPβ expression levels were assessed using a specific pre-validated assay obtained from Applied Biosystems. Total RNA from commercially obtained human SkM (BD Biosciences, Palo Alto, CA, USA) was reverse transcribed to cDNA and was used as the calibrator in all experiments. The ∆C_T_ value for each subject was expressed as a ratio with the ∆C_T_ measured from the commercially obtained SkM cDNA. Sufficient cDNA allowed quantification of the P1 and P2 promoter regions of *HSD11B1* before and after dexamethasone in 11/15 subjects.

### 11β-Hydroxysteroid dehydrogenase-1 enzyme activity

Oxoreductase enzyme activity of 11βHSD1 was determined in dispersed fresh SkM after overnight incubation, by measuring the conversion of radiolabeled ^3^H-cortisone to cortisol by thin layer chromatography, as described in our previous studies [5,26].

### Genomic DNA extraction

Muscle samples were incubated at 50°C overnight in DNA extraction buffer (100 mM NaCl, 10 mM Tris–HCl pH 8, 25 mM ethylenediamine tetraacetic acid, 0.5% SDS) with 200 μg/ml Proteinase K. DNA was then isolated by two rounds of phenol–chloroform extraction, followed by RNAse A treatment, precipitation in ethanol containing 10% sodium acetate (3 M, pH 5.2), with resuspension in 100 μl nuclease-free water (Ambion, Austin, TX, USA). DNA was quantitated and quality assessed by spectrophotometry on a Nanodrop (ThermoFisher Scientific, Waltham, MA, USA), and checked for integrity by gel electrophoresis, with subsequent storage at −20°C.

### *HSD11B1* promoter methylation

Figure 1 outlines the location of the *HSD11B1* promoter methylation assays used in this study. Sequenom MassARRAY EpiTYPING was carried out as previously described [28,29]. DNA samples were processed using the Methyl Easy bisulphite modification kit (Human Genetic Signatures, North Ryde, NSW, Australia) according to the manufacturer’s instructions. Amplification was performed on converted genomic DNA using primers directed to modified DNA: 11HSDB1_F1, 5^′^-aggaagagagTGGTGAAAAGGGAAAATTTGTTT and 11HSDB1_R1, 5^′^-cagtaatacgactcactatagggagaaggctCCTACAAAAACAACTCCAAAAAA for distal promoter 1 (P1); and 11HSDB1_F2, 5^′^-aggaagagagTTTTTGAAAGATTATTGATTTTTGG and 11HSDB1_R2, 5^′^-cagtaatacgactcactatagggagaaggctTCCTATAAAACACACAAAAAAAACC for proximal promoter 2 (P2) (lowercase denotes sequence tags added to facilitate downstream EpiTYPER analysis). EpiTYPER analysis produces a methylation β-value (approximately equivalent to total percentage methylation). All PCR amplifications and subsequent MassArray runs were performed in triplicate for calculation of mean methylation β-values at each analyzable CpG site.

### Statistical analysis

The raw data failed to satisfy parametric assumptions, and therefore nonparametric tests were used in the analysis. Tabulated data are expressed as the median (25th to 75th percentile), while Figure 2 presents the mean ± standard error of the mean. The mRNA expression, 11βHSD1 enzyme activity and changes in promoter DNA methylation score in response to dexamethasone were analyzed by the Wilcoxon signed-rank test, which examines the significance of the median difference between paired values. The correlation between the incremental response of 11βHSD1 activity and changes in mean promoter methylation was analyzed by the Spearman correlation coefficient. The level of statistical significance was set at *P* <0.05.

## Abbreviations

11βHSD1: 11β-hydroxysteroid dehydrogenase-1; C/EBP: CCAAT/enhancer-binding protein transcription factors; DMEM: Dulbecco’s Modified Eagle Medium; GRE: Glucocorticoid response element; PCR: Polymerase chain reaction; RT: Reverse transcriptase; SkM: Skeletal muscle.

## Competing interests

The authors declare that they have no competing interests.

## Authors’ contributions

WJI performed the statistical analysis and wrote the initial draft of the manuscript. VRO performed the biochemical, molecular and epigenetic analyses and assisted in editing the manuscript. CJ recruited the participants, performed the muscle biopsies and assisted in editing the manuscript. RS provided the intellectual expertise, coordinated epigenetic studies and assisted in manuscript preparation. All authors read and approved the final manuscript.
